# The association between serum vitamin D and obstructive sleep apnea: an updated meta-analysis

**DOI:** 10.1186/s12931-020-01554-2

**Published:** 2020-11-09

**Authors:** Xiaoyan Li, Jie He, Jie Yun

**Affiliations:** 1 Department of Endocrinology, Clinical Medical College and The First Affiliated Hospital of Chengdu Medical College, Chengdu, 610500 Sichuan China; 2 Department of Respiratory and Critical Care Medicine, Clinical Medical College and The First Affiliated Hospital of Chengdu Medical College, 278 Baoguang Street, Chengdu, 610500 Sichuan China; 3 Nursing Department of Affiliated Hospital of Chengdu University of Traditional Chinese Medicine, Chengdu, 610072 Sichuan China

**Keywords:** Vitamin D, Obstructive sleep apnea, Systematic review, Meta-analysis

## Abstract

**Background:**

The objective was to determine whether OSA patients have a low serum vitamin D level by systematic review and meta-analysis.

**Methods:**

This study searched the following electronic bibliographic databases: Embase, Medline, Web of Science, PubMed, VIP, Wanfang, CNKI and SinoMed. All data were searched between January 2000 and August 2020. The quality of the included studies was estimated by two researchers according to the Newcastle–Ottawa Scale and Agency for Healthcare Research and Quality. All qualified studies and statistical analyses were conducted using RevMan 5.2.

**Results:**

Twenty-nine eligible studies compromising 6717 participants met the inclusion criteria of the meta-analysis. The results revealed that the serum 25(OH)D level was significantly lower in OSA patients than the controls. According to the severity of the disease, subgroup analysis was performed; the results demonstrated that the serum 25(OH)D level was not decreased in mild OSA patients compared with the controls, while the serum 25(OH)D level in moderate and severe OSA patients was lower than that in the controls. Furthermore, based on ethnicity, BMI, PSG type, study quality and latitude, the subjects were divided into different subgroups for meta-analysis. The results revealed that the serum 25(OH)D level in all OSA subgroups was decreased compared with that in the control group.

**Conclusions:**

The present meta-analysis shows that the serum vitamin D level was different between OSA patients and healthy people. OSA patients could have a low serum vitamin D level.

## Background

Obstructive sleep apnea (OSA) refers to a sleep-related disorder characterized by repetitive, incomplete or total obstruction of the upper respiratory tract combined with hypopnea and apnea during sleep, contributing to an intermittent decrease in the partial pressure of blood oxygen and blood oxygen saturation and hypercapnia [1]. It is usually correlated with sleepiness, fatigue, inattention, memory loss, or even headaches during the day, seriously affecting quality of life and life expectancy [2]. Currently, polysomnography (PSG) is the gold standard for the diagnosis of OSA [3]. Patients with OSA have a greater risk of cardiovascular, cerebral, pulmonary vascular complications and even systemic multisystem pathophysiological changes [4, 5]. As an independent risk factor for many systemic diseases, OSA is currently remarkably prevalent in Western countries; therefore, some countries have listed it as a major health problem. The prevalence rate of OSA in Western countries is 2%–5% and is gradually increasing annually; the rate reached 43.1% in Iceland in 2016 among those aged 40 to 65 [6]. The incidence rate of OSA in adults in China is approximately 4%, according to an epidemiological survey [7].

It is well known that vitamin D is a liposoluble vitamin present in two primary patterns: D2 (ergocalciferol, gained from dietary resources) and D3 (cholecalciferol, generated in the skin under irradiation by ultraviolet light, or 25(OH)D) [8]. The main test to measure the serum vitamin D level is enzyme-linked immunosorbent assay (ELISA). Mass spectrometry (MS) is commonly used as well.

The relationship between 25-hydroxyvitamin D (25(OH)D) and OSA has been evaluated in several studies, including randomized controlled trials and observational studies [9–11]. Kerley et al. [9] discovered that 25(OH)D was markedly lower in OSA groups than in non-OSA groups, particularly in Caucasian populations. Mete et al. [10] declared that those with severe OSA had an elevated exposure to vitamin D deficiency. However, Yassa et al. [11] held that the severity of OSA was not related to serum vitamin D and that the vitamin D status did not alter the severity of OSA. While changes in the serum vitamin D level in patients with OSA may be relevant to inflammatory reactions, oxidative stress, energy metabolism, neuroendocrine regulation and so on, its specific pathogenesis is poorly understood.

Until now, the viewpoint that OSA patients have lower serum vitamin D has remained controversial. Thus, a meta-analysis in 2018 [12] comparing the 25(OH)D serum level between controls and OSA patients was conducted. Whereas the main locations of the included studies are developed countries, such as Europe and North America; additionally, a former study did not compare the 25(OH)D serum level according to OSA severity. Therefore, this conclusion remains to be verified in observational studies. Recently, continuous research has been carried out to confirm whether low serum levels of vitamin D are pervasive in those with OSA, especially those with serious OSA. New research on OSA in Asians has emerged, but the results are contradictory. Thus, the objective of our updated meta-analysis is to include the latest observational studies in determining whether different types of OSA patients exhibit low serum vitamin D.

## Materials and methods

### Search strategy

Our meta-analysis was registered at the prospective register of systematic reviews (PROSPERO, the website is https://www.crd.york.ac.uk/PROSPERO/, and the ID is CRD42020172659). The following eight electronic databases were searched individually from January 2000 to August 2020: Embase, Medline, Web of Science, PubMed, VIP, Wanfang, CNKI and SinoMed. The search terms were [(“vitamin D”) or (“vit D”) or (“ergocalciferol”) or (“cholecalciferol”)] and [(“sleep”) or (“obstructive sleep apnea”) or (“sleep apnea syndromes”) or (“obstructive sleep apnea syndromes”)].

### Study selection and exclusion criteria

The literature included met the following standards: (1) any observational study design, including case–control, cross-sectional and cohort studies; (2) literature reporting on the vitamin D level in OSA patients without restrictions on age, ethnicity, nationality or sex; (3) OSA diagnosed by polysomnography (PSG); and (4) search limited to full articles in English and Chinese. Studies meeting the following criteria were excluded: (1) the type of research was a case report, review, letter, commentary or editorial; and (2) the article did not contain adequate data, and it was difficult to contact the authors to obtain valid sources.

### Study selection

The title and abstract of internationally relevant studies were screened by two review authors individually on the basis of our search strategy. The full text of the study was attained and confirmed if the abstract was eligible. Then, we reappraised potentially suitable studies by retrieving and assessing the full text. If there was conflict about the inclusion or exclusion of a study, the disagreement between reviewers was settled by analysis or consultation with the third reviewer.

### Data extraction and management

Two reviewers (X.Y.L. and J.H.) obtained the data independently. A designed table was used to extract data from each included paper, as follows (1) basic information of the paper: study design, country, year of publication, first author, etc.; (2) baseline characteristics of the participants: age, number and sex; (3) OSA measurement method and its type; and (4) study quality. If a study eligible for our meta-analysis lacked essential content, the authors were contacted by telephone or e-mail no less than twice. Then, authors were requested to supply the missing data.

### Methodological quality assessment

The methodological quality evaluation of all studies was conducted by two researchers independently. If a disagreement occurred in the process of the quality evaluation, it was negotiated by two reviewers or arbitrated by a specialist in the field. Cohort and case–control studies were evaluated through the Newcastle–Ottawa Scale (NOS) [13], which included study population selection (4 items, full score 4), comparability (1 item, full score 2), and exposure or outcome (3 items, full score 3); scores of 0–3, 4–6 and 7–9 were considered to indicate low- (grade C), medium- (grade B) and high-quality (grade A) studies, respectively. Cross-sectional studies were evaluated using the quality evaluation criteria recommended by the Agency for Healthcare Research and Quality (AHRQ) [14], which includes 11 items. Each item was rated as "yes", "no", or "unclear", with a score of 1 for "yes" and 0 for "no" or "unclear". Scores of 0–3, 4–7 and 8–11 were considered to indicate low- (grade C), medium- (grade B) and high-quality (grade A) studies, respectively.

### Statistical analysis

A meta-analysis was performed to assess the summarized results of the studies with RevMan 5.3 software. Binary variables are expressed by odds ratios (ORs) and 95% confidence intervals (CIs), and continuous variables are expressed by standard mean differences (SMDs) and 95% CIs. Chi-square tests of Cochrane's Q statistic and I-squared were used to analyze the heterogeneity of the results, with heterogeneity thresholds of low (25%), moderate (50%), and high (75%). If P > 0.1 and I^2^ < 50% indicated homogeneity among studies, a fixed-effects model was adopted; if P ≤ 0.1 and I^2^ ≥ 50% revealed heterogeneity, a random-effects model was used. Obvious heterogeneity was analyzed by subgroup analysis or meta-regression; if more than one article existed in the subgroup, the analysis was performed by sex, country, number of samples, study design, study quality and severity of OSA (the apnea–hypopnea index (AHI) was applied in the classification; an AHI of 5–14.9, 15–29.9 and ≥ 30 events/hour was considered to indicate mild, moderate and severe OSA, respectively). Publication bias was examined in testing the results of the quantitative evaluation.

## Results

### Literature search and included studies

**Fig. 1:**
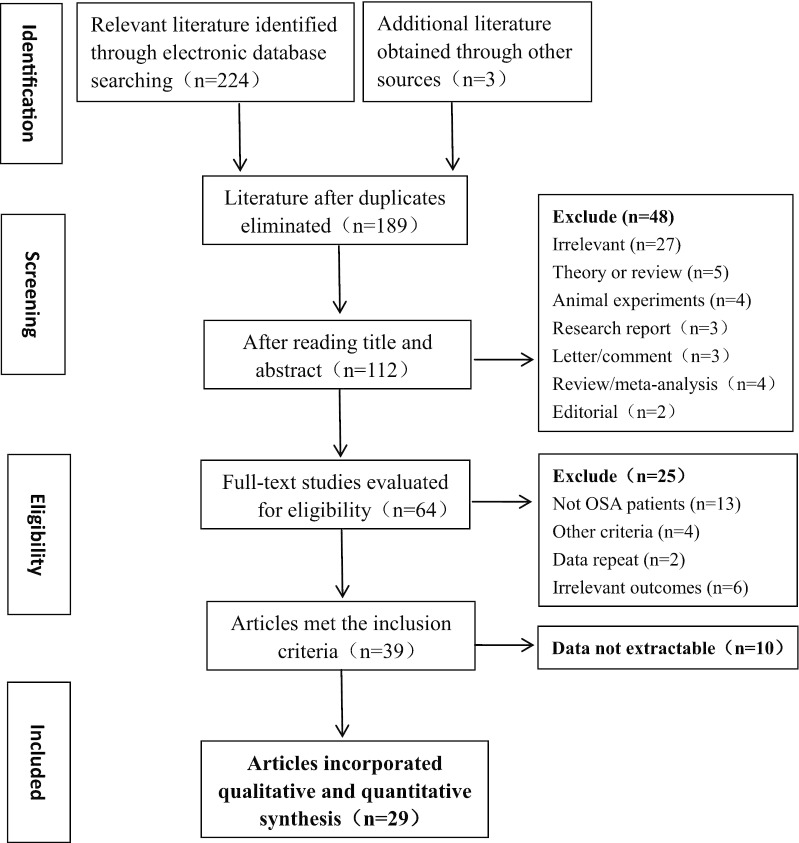
Flow diagram of literature selecting process and results according to the preferred reporting items for the meta-analysis. OSA: obstructive sleep apnea

The primary yield from the databases was 227 relevant studies. There were 189 potentially eligible studies, and some repetitive studies were removed later. The quantity was reduced to 112 after assessing titles and abstracts, and then 48 articles were excluded. The 64 remaining studies were downloaded, and the full text was screened according to the well-established inclusion and exclusion standards described here. Finally, the remaining 29 articles [9–11, 15–40] were eligible for analysis. A flow diagram of the literature selection process and results is presented (Fig. 1).

The 29 included studies involved 6717 participants (Table 1). There were 6 case–control studies, 21 cross-sectional studies and 2 cohort studies. Among them, 22 English articles comprising 6043 cases and 7 Chinese articles involving 674 cases were included. In all studies, OSA was diagnosed by polysomnography (PSG). The vitamin D level was evaluated by measuring the serum 25(OH)D level by ELISA, electrochemiluminescence and MS.

**Table 1:** Characteristics and quality/degree of these included paper concerning the association between vitamin D and OSA

Author, Year	Country	Study design	Male	Ages/n	Sample number					Measurement and type	Quality/Degree
					Control	Mild OSA	Moderate OSA	Severe OSA	Any OSA		
Bozkurt 2012 [15]	Altindag,Turkey	CSS	59.5%	49.7 ± 9.8	47	46	47	50	143	PSG I	8/A
Mete 2013 [10]	Ankara, Turkey	CSS	50%	47.1 ± 8.6	32	50	50	50	150	PSG I	6/B
*Kheirandish-Gozal 2014 [16]	Chicago,USA	CSS	52.8%	6.8 ± 0.8	74	NA	NA	NA	102	PSG I	8/A
Erden 2014 [17]	Hatay, Turkey	CSS	71.1%	47.4 ± 11.9	43	NA	23	62	85	PSG I	9/A
Liguori 2015 [18]	Rome, Italy	CCS	67.2%	60.6 ± 11.7	32	NA	NA	90	90	PSG I	8/A
Yang 2015 [19]	Shanghai, China	CSS	75%	62.5 ± 8.6	36	NA	NA	NA	36	PSG I	7/A
Goswami 2016 [20]	Minnesota, USA	CS	100%	76.4 ± 5.5	1104	977	470	276	1723	PSG I	8/A
Kerley 2016 [9]	Dublin, Ireland	CSS	68.9%	54.5(median)	31	22	18	35	75	PSG I	8/A
Zicari 2016 [21]	Rome, Italy	CSS	66%	8.5 ± 2.2	70	NA	NA	NA	22	PSG I	8/A
Klobucnikova 2017 [22]	Bratislava, Slovakia	CSS	73.6%	56.3 ± 11.9	55	NA	NA	NA	55	PSG I	7/A
Toujani 2017 [23]	Tunis, Tunisia	CSS	54.1%	52.3 ± 12.7	30	NA	NA	92	92	PSG I	8/A
Salepci 2017 [24]	Istanbul,Turkey	CSS	60.2%	49 ± 12	19	53	38	71	162	PSG I	9/A
Archontogeorgis 2017[25]	Alexandroupolis, Greece	CCS	79.9%	52.3 ± 13.2	30	NA	NA	NA	139	PSG I	8/A
Liguori 2017 [26]	Rome, Italy	CCS	NA	NA	10	NA	NA	NA	39	PSG I	8/A
Zhang 2017 [27]	Fujian, China	CSS	91.2%	45.6 ± 12.2	39	32	21	33	86	PSG II	6/B
Zhu 2017 [28]	Shanghai, China	CSS	51.9%	46.5 ± 12.8	38	38	46	36	120	PSG III	6/B
Liu CY 2017 [29]	Jiangsu,China	CSS	47.6%	49.9 ± 5.1	20	NA	NA	NA	22	PSG III	5/B
Liu T 2017 [30]	Shandong, China	CSS	65.8%	5.9 ± 2.3	70	NA	NA	NA	47	PSG II	8/A
Archontogeorgis 2018 [31]	Alexandroupolis, Greece	CCS	82.2%	54.8 ± 12.4	52	NA	NA	NA	55	PSG I	7/A
Han 2018 [32]	Liaoning, China	CCS	55%	43.2 ± 6.8	40	13	12	15	40	PSG III	5/B
Ou 2019 [33]	Hunan, China	CSS	51.1%	59.7 ± 13.2	40	NA	NA	NA	40	PSG II	4/B
Fan 2019 [34]	Guangdong, China	CSS	88.4%	27–79	8	18	33	53	104	PSG II	8/A
Ragia 2019 [35]	Alexandroupolis, Greece	CCS	80.1%	52.2 ± 12.9	32	NA	NA	NA	144	PSG I	7/A
Kirac 2019 [36]	Istanbul,Turkey	CSS	46%	47.3 ± 9.9	50	NA	NA	NA	50	PSG I	5/B
Archontogeorgis 2019 [37]	Alexandroupolis, Greece	CCS	88%	50 ± 11.7	34	NA	NA	NA	58	PSG I	8/A
Yassa 2019 [11]	Bartin, Turkey	CSS	81.4%	46.8 ± 11.3	60	NA	NA	NA	61	PSG I	4/B
Bouloukaki 2020 [38]	Crete, Greece	CSS	74%	54 ± 15	68	94	150	373	617	PSG I	8/A
Siachpazidou 2020 [39]	Larissa,Greece	CS	63.3%	53.2 ± 11.6	30	NA	NA	NA	30	PSG I	7/A
Ma 2020 [40]	Fujian, China	CSS	66.2%	49 ± 14	30	33	31	42	106	PSG I	8/A

*CSS* cross sectional study, *CCS* case–control study, *CS* cohort studies, *OSA* obstructive sleep apnea, *NA* not applicable, *PSG* polysomnogram;

A: grade A; B: grade B; C: grade C

As a supplementary explanation, one article (Kheirandish-Gozal 2014) [16] was admitted to the systematic review but excluded from the meta-analysis because the data were too high to adopt, as explained in the previous meta-analysis [12]. Overall, there were 29 studies in the systematic review and 28 articles in the meta-analysis.

### Quality assessment

According to the NOS [13] and AHRQ [14], this study assessed the methodological quality of all studies (Table 1). The overall quality of the included studies was relatively high depending on the evaluation level; 21 studies were grade A, and 8 were grade B.

### Main results–vitamin D level in all OSA patients

**Fig. 2:**
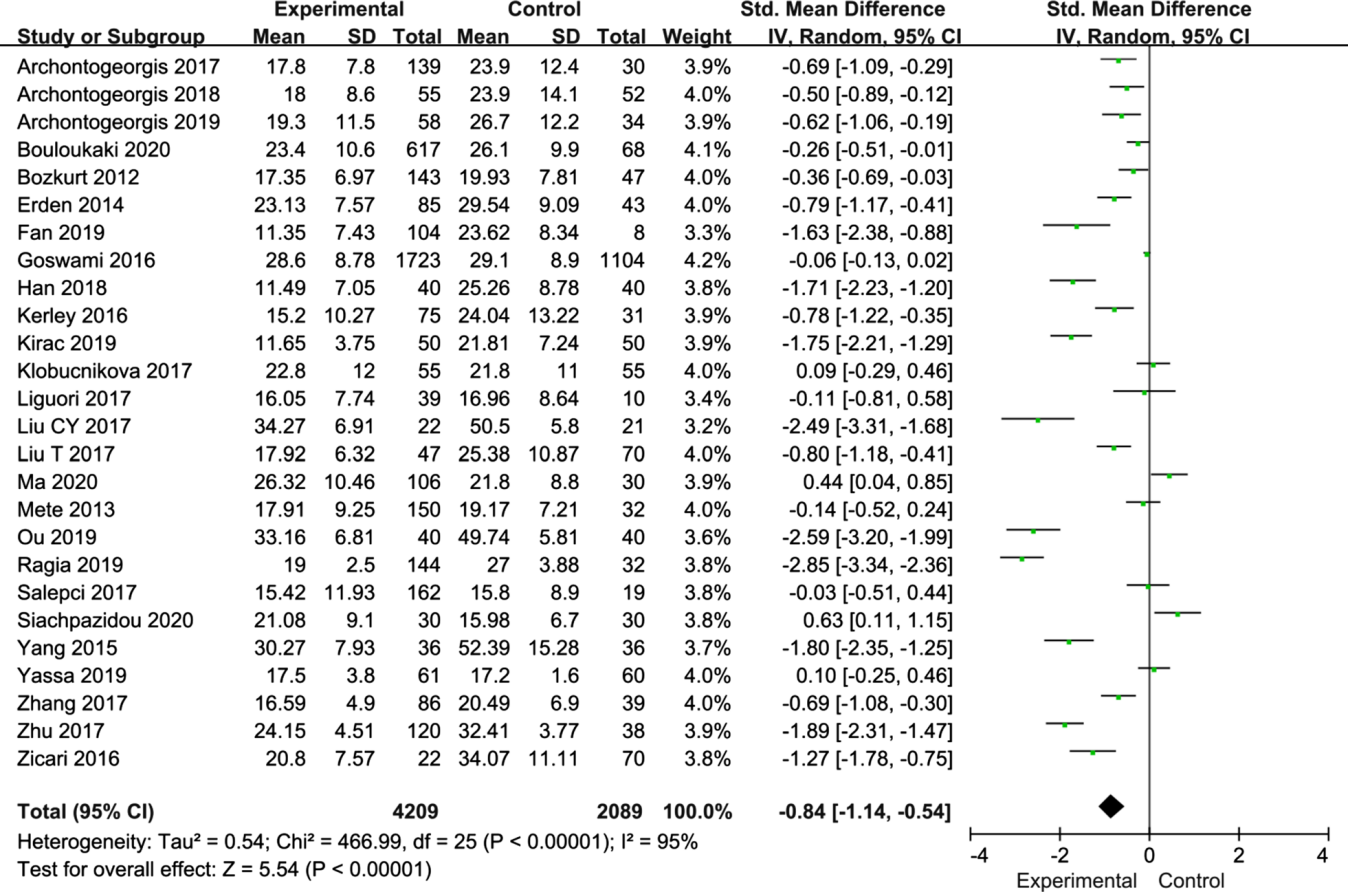
Forest plot of SMD and its 95%CI for serum 25(OH)D levels in integral OSA patients group compared to the control group in meta-analysis. SMD: standard mean difference; CI: confidence interval; OSA: obstructive sleep apnea

Among the included publications, twenty-six articles reported vitamin D level in all OSA patients. Due to the high heterogeneity (I^2^ = 95%), we chose a random-effects model to analyze all of the studies. The meta-analysis results revealed that the serum 25(OH)D level was significantly lower in OSA patients than in the controls (SMD = − 0.84; 95% CI, − 1.14–0.54; P < 0.00001) (Fig. 2).

### Subgroup analysis

Some of the included articles reported the vitamin D levels in patients with OSA of different severities, so we carried out a subgroup analysis based on mild, moderate and severe OSA. The remaining subgroup analysis was conducted according to the vitamin D levels in all OSA patients.

### OSA severity

**Fig. 3:**
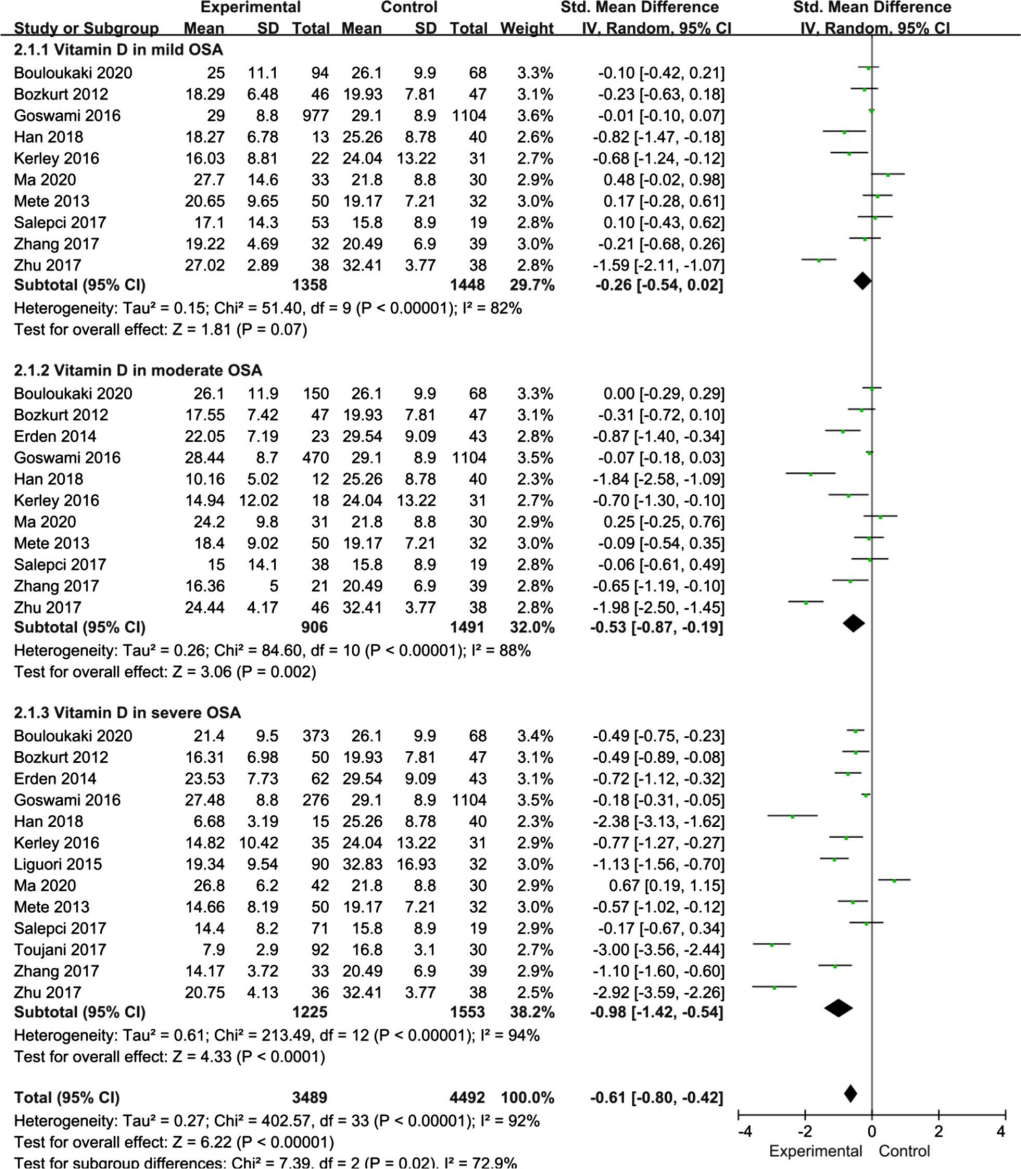
Subgroup analysis depended on different severity of OSA patients compared to the controls was displayed in meta-analysis. The means of expression are SMD and its 95%CI. SMD: standard mean difference; CI: confidence interval; OSA: obstructive sleep apnea

Fortunately, most relevant studies have reported the relationship between the severity of OSA and the serum 25(OH)D level compared with the control. Thus, in the current meta-analysis, OSA patients were stratified into those with mild OSA, moderate OSA and serious OSA based on the AHI. Ten studies provided data on the serum 25(OH)D level in mild OSA. The present study adopted a random-effects model for subgroup analysis. The results revealed that the serum 25(OH)D level in mild OSA patients was not significantly different from that in the controls (SMD = − 0.26; 95% CI = − 0.54, 0.02; P = 0.07; I^2^ = 82%) (Fig. 3). Eleven studies provided data on the serum 25(OH)D level in controls and patients with moderate OSA. The serum 25(OH)D level in moderate OSA patients was lower than that in the controls (SMD = − 0.53; 95% CI = − 0.87, − 0.19; P = 0.002; I^2^ = 88%) (Fig. 3). Thirteen studies provided data on the serum 25(OH)D level in controls and patients with severe OSA. Interestingly, the serum 25(OH)D level in severe OSA patients was lower than that in the controls (SMD = − 0.98; 95% CI = − 1.42, − 0.54; P < 0.0001; I^2^ = 94%) (Fig. 3).

### Ethnicity

Subgroup analysis can be used to explain the cause of heterogeneity. According to ethnicity, the subjects were divided into Caucasian and Asian populations. In the Caucasian group (involving 5,254 patients from 16 studies), the serum 25(OH)D level in the case group was lower than that in the healthy control group (SMD = − 0.59; 95% CI = − 0.9, − 0.27; P = 0.0003; I^2^ = 93%) (Table 2). Similarly, in the Asian group (involving 1044 patients from 10 studies), the serum 25(OH)D level in the case group was lower than that in the healthy control group (SMD = − 1.28; 95% CI = − 1.92, − 0.64; P < 0.0001; I^2^ = 94%) (Table 2).

**Table 2:** Subgroup analyses of the association between vitamin D and OSA in this meta-analysis

Subgroup	No. of studies	Sample		Mean difference (95% CI)	P for Heterogeneity	I-squared Value	P Value between groups
		Experiment	Control				
Ethnic							
Caucasian	16	3547	1707	− 0.59[− 0.9, − 0.27]	0.0003	93%	P < 0.00001
Asian	10	662	382	− 1.28[− 1.92, − 0.64]	P < 0.0001	94%	P < 0.00001
BMI							
Normal range	3	176	129	− 1.66[− 2.73, − 0.59]	0.002	93%	P < 0.00001
Overweight	4	1983	1186	− 1.33[− 2.55, − 0.1]	0.03	98%	P < 0.00001
Obesity	17	1981	634	− 0.57[− 0.93, − 0.21]	0.002	92%	P < 0.00001
PSG degree							
I	19	3750	1833	− 0.55[− 0.85, − 0.26]	0.0002	93%	P < 0.00001
II	4	277	157	− 1.4[− 2.21, − 0.58]	0.0008	91%	P < 0.00001
III	3	182	99	− 1.93[− 2.28, − 1.58]	P < 0.00001	21%	0.28
Study quality							
High/A	18	3640	1769	− 0.61[− 0.92, − 0.31]	P < 0.0001	93%	P < 0.00001
Medium/B	8	569	320	− 1.37[− 2.07, − 0.66]	0.0001	95%	P < 0.00001
Study latitude							
Low latitude	4	336	117	− 1.24[− 2.25, − 0.23]	0.02	94%	P < 0.00001
Middle latitude	22	3873	1972	− 0.8[− 1.11, − 0.49]	P < 0.00001	95%	P < 0.00001

*OSA* Obstructive sleep apnea, *BMI* body mass index, *PSG* polysomnogram

### Body mass index (BMI)

Most of the included studies reported BMI data. While two reported BMI in children, which differs from adult standards, we performed a meta-analysis of the remaining 24 studies according to the classification of normal, overweight and obese adults. Three studies provided data on the serum 25(OH)D level in OSA patients with a normal BMI, and the results revealed that the serum 25(OH)D level was lower in these OSA patients than in the controls (SMD = − 1.66; 95% CI = − 2.73, − 0.59; P = 0.002; I^2^ = 93%) (Table 2). Four studies provided data on the serum 25(OH)D level in overweight OSA patients, and the results revealed that the serum 25(OH)D level was significantly lower in overweight OSA patients than in the controls (SMD = − 1.33; 95% CI = − 2.55, − 0.1; P = 0.03; I^2^ = 98%) (Table 2). Seventeen studies provided data on the serum 25(OH)D level in obese OSA patients, and the results revealed that the serum 25(OH)D level was significantly lower in obese OSA patients than in the controls (SMD = − 0.57; 95% CI = − 0.93, − 0.21; P = 0.002; I^2^ = 92%) (Table 2).

### PSG type

We carried out a subgroup analysis according to the type of PSG equipment used for diagnosing OSA. Nineteen studies provided data on the serum 25(OH)D level in OSA patients diagnosed by type I PSG, and the results revealed that the serum 25(OH)D level was lower in these OSA patients than in the controls (SMD = − 0.55; 95% CI = − 0.85, − 0.26; P = 0.0002; I^2^ = 93%) (Table 2). Four studies provided data on the serum 25(OH)D level in OSA patients diagnosed by type II PSG, and the results revealed that the serum 25(OH)D level was lower in these OSA patients than in the controls (SMD = − 1.4; 95% CI = − 2.21, − 0.58; P = 0.0008; I^2^ = 91%) (Table 2). Three studies provided data on the serum 25(OH)D level in OSA patients diagnosed by type III PSG, and the results revealed that the serum 25(OH)D level was lower in these OSA patients than in the controls (SMD = − 1.93;95% CI = − 2.28, − 1.58; P < 0.00001; I^2^ = 21%) (Table 2).

### Study quality

Considering that variations in the quality of the literature would affect the heterogeneity of the effect, we carried out a corresponding subgroup analysis. Eighteen high-quality/grade A studies provided data on the serum 25(OH)D level in OSA patients, and the results revealed that the serum 25(OH)D level was lower in the OSA patients than in the controls (SMD = − 0.61; 95% CI = − 0.92, − 0.31; P < 0.0001; I^2^ = 93%) (Table 2). Eight moderate-quality/grade B studies provided data on the serum 25(OH)D level in OSA patients, and the results revealed that the serum 25(OH)D level was lower in OSA patients than in the controls (SMD = − 1.37; 95% CI = − 2.07, − 0.66; P = 0.0001; I^2^ = 95%) (Table 2).

### Study latitude

Light duration varies at different latitudes, which may affect the serum 25(OH)D level in OSA patients. Areas with latitude values of 0–30, 30–60, and 60–90 degrees were considered low-latitude, mid-latitude, and high-latitude areas, respectively. Four studies provided data on the serum 25(OH)D level in OSA patients in low-latitude areas, and the results revealed that the serum 25(OH)D level was lower in these OSA patients than in the controls (SMD = − 1.24; 95% CI = − 2.25, − 0.23; P = 0.02; I^2^ = 94%) (Table 2). Twenty-two studies provided data on the serum 25(OH)D level in OSA patients in mid-latitude areas, and the results revealed that the serum 25(OH)D level was lower in these OSA patients than in the controls (SMD = − 0.8; 95% CI = − 1.11, − 0.49; P < 0.00001; I^2^ = 95%) (Table 2).

### Meta-regression and sensitivity analyses

Examination of the association between the serum 25(OH)D level and OSA in all patients revealed high heterogeneity (I^2^ = 95%, P < 0.00001). Therefore, we carried out a meta-regression to determine the sources of the high heterogeneity. The meta-regression indicated P values of 0.168, 0.247, 0.138, 0.452, 0.278, and 0.092 for covariates of ethnicity, disease severity, PSG type, study quality, BMI and latitude, respectively. This information suggests that the former factors had no significant effect on heterogeneity. After sequentially excluding each study, we failed to find any study that could have an effect on the final outcome. The sensitivity analysis showed that this meta-analysis was stable.

### Publication bias

**Fig. 4:**
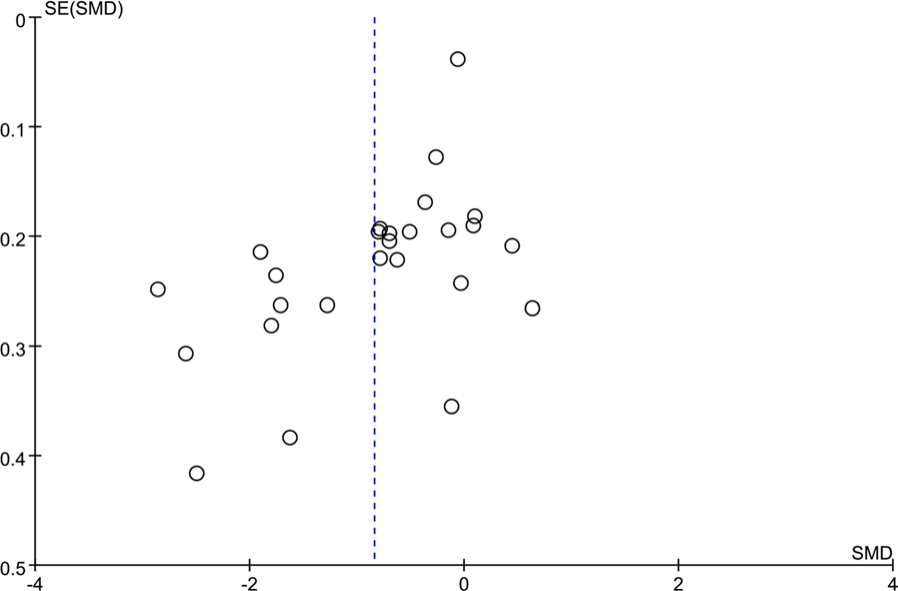
Funnel plot was to evaluate the publication bias among those included literatures about the association between serum 25(OH)D levels and OSA. SMD: standard mean difference; OSA: obstructive sleep apnea

A funnel plot was used to evaluate the publication bias among the included studies regarding the association between the serum 25(OH)D level and OSA. The results revealed no noticeable publication bias (Fig. 4).

## Discussion

Vitamin D deficiency has been noted in many metabolic diseases, and it is thought to be related to OSA progression [21]. The present meta-analysis assessed the serum 25(OH)D level in OSA patients. The combined results show that the serum 25(OH)D level was noticeably lower in OSA patients than in the controls. Moreover, with increasing OSA severity, the serum 25(OH)D level decreased more obviously, suggesting that serum 25(OH)D might be a risk factor for OSA. Quite notably, the decrease in the serum 25(OH)D level was more obvious in Asians than in Caucasians. This phenomenon suggests that ethnicity may also affect the serum 25(OH)D level in OSA patients. Differences in the level of serum 25(OH)D among different ethnicities might be related to polymorphisms in vitamin D receptor genes and metabolic genes. Kirac et al. [36] found that VDR (rs2228570) and VDBP (rs4588 and rs7041) mutations were highly related to OSA in Caucasian populations. Nie et al. [41] indicated that OSA was associated with the angiotensin-converting enzyme gene, which plays a crucial role in vitamin D metabolism in Asian populations. Moreover, the serum 25(OH)D level decreased with increasing OSA severity. Both overweight OSA patients and obese OSA patients had low levels of vitamin D, indicating that BMI and OSA interact to influence the vitamin D level. A high BMI and OSA are causally interrelated. One study confirmed that low circulating levels of vitamin D were associated with obesity in humans, and obesity was thought to be one of the reasons for the reduction in the 25(OH)D level [42]. Furthermore, subgroup analyses were conducted according to the PSG type, study quality and latitude, and the serum 25(OH)D level was still lower in the OSA patients. The degree of decline in the serum 25(OH)D level varied based on the above three factors. Of course, these differences may have been caused by individual differences among OSA patients and various detection instruments. However, overall, the results of the meta-analysis are genuine and reliable.

The underlying mechanism of the relationship between the serum 25(OH)D concentration and OSA remains unclear [11]. According to the source, metabolism and various influencing factors of vitamin D [26, 43, 44], we have summarized several seemingly reasonable biological explanations. Previous studies have proven that there is a significant correlation between the vitamin D level and obesity, and most OSA patients are obese [21]. Most OSA patients included in our study had a BMI greater than 30, which meets the diagnostic criteria of obesity. Some obese patients usually do not like outdoor activities. Lack of outdoor activity might result in reduced vitamin D synthesis due to insufficient sun exposure. In addition, vitamin D, as a fat-soluble vitamin, is stored by adipose tissue [45]. In obese patients, who have greater amounts of adipose tissue, the storage distribution volume of vitamin D is also remarkably increased [45]. This effect of increased vitamin D storage in adipose tissue reduces the release of vitamin D into the circulation, resulting in a lower bioavailability of vitamin D. Fan et al. [34] reported that vitamin D was negatively correlated with BMI and that there was an interaction between the vitamin D level and obesity. Moreover, 1 h of sleep disorder can reduce daytime activity by 3%, and with the loss of 1 h of sleep, the probability of obesity increases by 80%. Due to lack of sleep or poor sleep quality, OSA aggravates obesity, forming a vicious circle. Consequently, the vitamin D level becomes significantly lower in obese OSA patients.

Many factors can contribute to vitamin D deficiency. In addition to obesity, geographical location and solar exposure also have influences [46]. Theoretically speaking, OSA patients at low latitudes near the equator should have higher levels of vitamin D, but people near the equator tend to have darker skin, and skin melanin reduces the ability of the skin to synthesize vitamin D. Neighbors et al. [12] evaluated the latitude of the study and the vitamin D level in the study population and found no significant correlation between the latitude and serum 25(OH)D level. In this meta-analysis, the serum 25(OH)D level was lower in OSA patients than in the controls, regardless of whether the patients were from low- or mid-latitude areas. The degree of reduction in the serum 25(OH)D level was similar between low- and mid-latitude areas. Therefore, geographical location exerted little influence on the overall results of this study.

Some studies have reported that sleep fragmentation in patients with OSA due to nocturnal hypoxia can lead to daytime drowsiness, fatigue and other symptoms. Sleep fragmentation can lead to a decrease in outdoor activity and a decrease in vitamin D synthesis [47]. In addition, due to the repeated aggravation of upper airway obstruction and hypopnea in patients with OSA, autonomic nerve function is mainly increased in terms of sympathetic nerve activity at night, and there is abnormal tension of the vagus nerve [48]. Excitation of the sympathetic nervous system could partly inhibit vagus nerve activity, while abnormal vagus nerve activity affects gastrointestinal motility and the secretion of gastrointestinal hormones, in turn affecting the absorption and metabolism of vitamin D. At the same time, OSA patients suffer from sleep disturbances, intermittent hypoxia, upper airway obstruction and increased abdominal pressure for a long time [49]. All of these disorders could result in gastroesophageal reflux and gastric ischemia, which also affect the absorption of vitamin D [25]. Therefore, the level of serum vitamin D in patients with severe OSA is significantly lower than that in healthy controls.

Among the included studies, the study by Liu et al. [30] demonstrated that tonsillar hypertrophy was related to low serum vitamin D and that tonsillar hypertrophy is one of the main causes of OSA in children. The mechanism may be that vitamin D could be related to immune regulation [50–52]. A normal serum 25(OH)D level can enhance innate immunity, and a low level of vitamin D can significantly decrease the total number of peripheral T lymphocytes and the percentage of T cells, causing reduced cellular immunity and humoral immune function. Moreover, a low vitamin D level gives rise to dysfunctional upper airway immune regulation. Lower levels of vitamin D promote the production of inflammatory transmitters, such as tumor necrosis factor-α and interleukin-1, which increase the risk of respiratory tract infection [49, 53, 54]. This leads to tonsillar hypertrophy, chronic rhinitis and nonspecific myopathy, which may aggravate OSA in children.

Generally, heterogeneity among studies in meta-analyses is related to the quality of the included research, population characteristics, experimental methods and other factors. High heterogeneity was found among the studies on the relation between serum vitamin D and OSA in our study. To explore the underlying source of heterogeneity, subgroup and meta-regression analyses were performed. Meta-regression showed no heterogeneity with respect to ethnicity, disease severity, PSG type, study quality, BMI or latitude. Regardless of the meta-regression results, we still conducted subgroup analyses by ethnicity, disease severity, PSG type, study quality, BMI and latitude. Unfortunately, no evident sources of heterogeneity were found. However, these factors could also increase the heterogeneity and reduce the reliability of this meta-analysis. In addition, the "leave-one-out" sensitivity study revealed no individual studies contributing to the high heterogeneity. Thus, we speculated that there are several other factors leading to the heterogeneity, including differences in blood sampling and storage methods, measurement methods and experimental conditions, light duration and climate in different regions, dietary intake and other underlying confounding factors.

This meta-analysis has several strengths in the examining the vitamin D serum level in relation to OSA. First, the overall results suggest that the serum 25(OH)D level may be a clinically useful biological indicator, which may help clinicians objectively evaluate the severity of OSA and provide an improved understanding of the potential pathophysiology involved in OSA. Kerley et al. [55] pointed out that vitamin D3 supplementation could improve several physiological, biochemical and subjective features of OSA as well as decrease metabolic markers compared to a placebo. The effect size of the serum 25(OH)D level could suggest whether vitamin D supplementation is suitable in OSA patients. Nevertheless, the size of the study by Kerley et al. [55] was very small, and the clinical significance of the serum 25(OH)D level needs further evaluation in larger trials. Second, this is the largest meta-analysis of the relevant literature, and subgroup analyses were carried out to provide more robust results. We also included the most recent published studies involving Chinese populations in this meta-analysis. Third, all of the included articles were of medium or high quality, making the assessment here more feasible. Fourth, no obvious publication bias was detected, suggesting that the combined results may be reliable.

However, there are still a number of underlying limitations to the study. First, the inconsistencies in the serum sample detection methods and the dietary diversity of the population are factors of heterogeneity, which could result in statistical error. Second, since this study lacked effective longitudinal cohort studies, we could not infer causality of the association between OSA and serum vitamin D. Third, due to data limitations, dose–response relationships between serum vitamin D and OSA risk were not obtained.

## Conclusion

It is concluded that a low serum vitamin D level may be correlated with OSA and that OSA could be accompanied by a low serum vitamin D level. Finally, more elaborate studies are required in the future to ascertain the association of serum vitamin D with OSA risk.

## Data Availability

None.

## Abbreviations

OSA: Obstructive sleep apnea
NA: Not applicable
PSG: Polysomnogram
CSS: Cross sectional study
CCS: Case–control study
NOS: Newcastle–Ottawa Scale
AHRQ: Healthcare Research and Quality
AHI: Apnea–hypopnea index
BMI: Body Mass Index
